# Supplementary material for: Individual differences in behavioral effects of xylazine and opioid-xylazine mixtures in male rats
Source: bioRxiv. 2025 Jun 27:2025.06.25.661558. Preprint. [Version 1] doi: 10.1101/2025.06.25.661558 (PMC12262312; doi:10.1101/2025.06.25.661558)
Supplement: 1 [file NIHPP2025.06.25.661558V1-supplement-1.pdf]

bioRxiv preprint doi: https://doi.org/10.1101/2025.06.25.661558; this version posted June 27, 2025. The copyright holder for this preprint (which was not certified by peer review) is the author/funder, who has granted bioRxiv a license to display the preprint in perpetuity. It is made available under aCC-BY 4.0 International license.

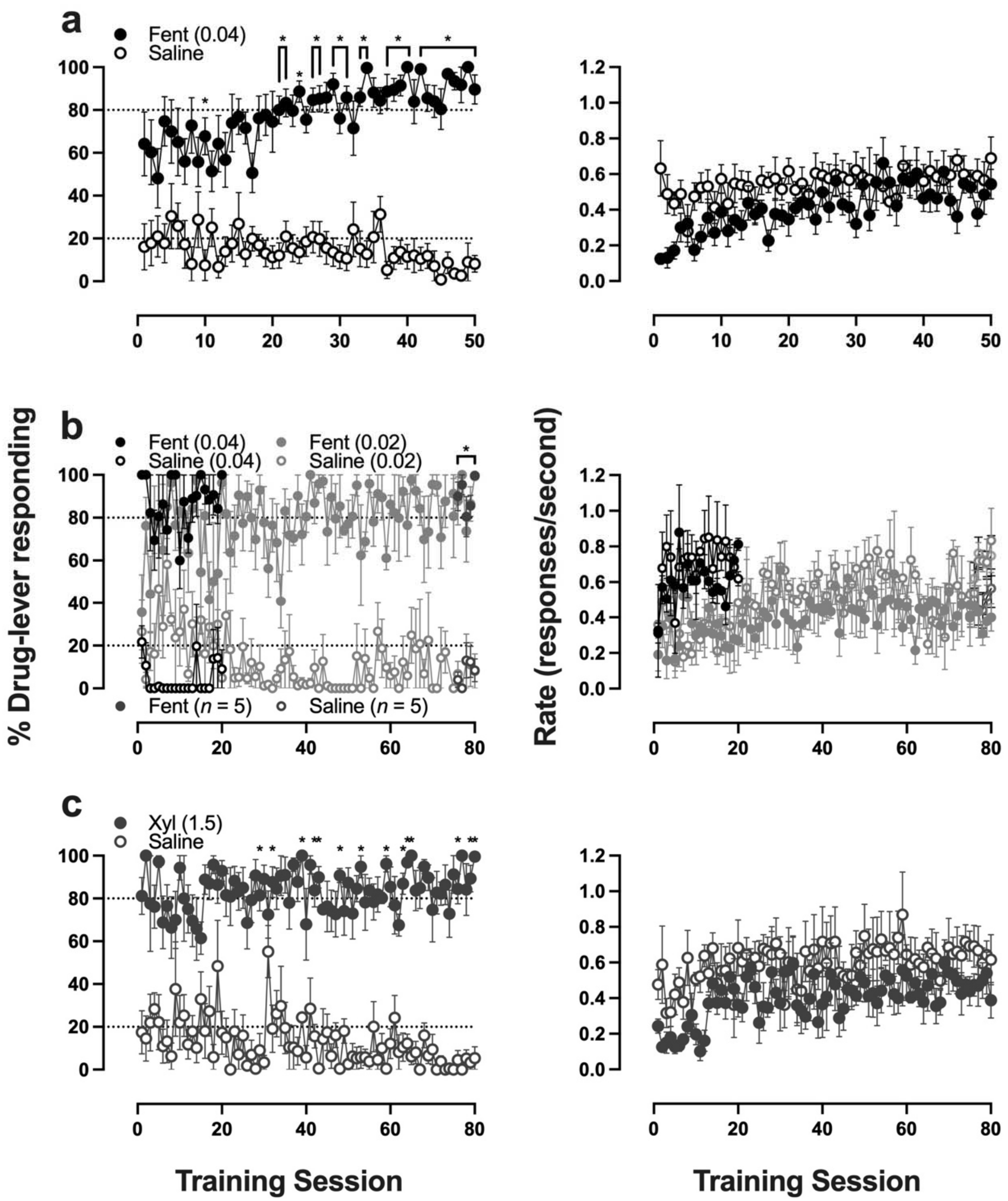

Supplemental Fig. 1 Acquisition curves of the discriminative stimulus effects of fentanyl or xylazine from vehicle in rats.
Percent responding on the drug-appropriate lever (*left*) and response rate (*right*) from the first active period during training sessions in rats trained to discriminate 0.04 mg/kg fentanyl (**a**), 0.02 mg/kg fentanyl (**b**), and 1.5 mg/kg xylazine (**c**). Asterisks indicate significant difference from saline sessions.
